# Author Correction: Structured pathways in the turbulence organizing recent oil spill events in the Eastern Mediterranean

**DOI:** 10.1038/s41598-023-31935-8

**Published:** 2023-03-23

**Authors:** Guillermo García‑Sánchez, Ana M. Mancho, Antonio G. Ramos, Josep Coca, Stephen Wiggins

**Affiliations:** 1 Instituto de Ciencias Matemáticas, CSIC, C/ Nicolás Cabrera 15, Campus Cantoblanco, 28049 Madrid, Spain; 2 Escuela Técnica Superior de Ingenieros de Telecomunicación, Universidad Politécnica de Madrid, 28040 Madrid, Spain; 3 Instituto ECOAQUA, Faculty of Marine Sciences, Campus Universitario de Tafira, Universidad de Las Palmas de Gran Canaria, 35017 Las Palmas de Gran Canaria, Spain; 4 School of Mathematics, University of Bristol, Bristol, BS8 1TW UK

Correction to: *Scientific Reports* 10.1038/s41598-022-07350-w, published online 07 March 2022

The original version of this Article omitted an affiliation for Guillermo García‑Sánchez. The correct affiliations are listed below.

Instituto de Ciencias Matemáticas, CSIC, C/ Nicolás Cabrera 15, Campus Cantoblanco, 28049 Madrid, Spain.

Escuela Técnica Superior de Ingenieros de Telecomunicación, Universidad Politécnica de Madrid, 28040 Madrid, Spain.

Furthermore, the Supplementary Information file(s) contained an error, where the title of the original Article in these files was incorrect.

The original Article and accompanying Supplementary Information file have been corrected.

