# Supplementary material for: Structured pathways in the turbulence organizing recent oil spill events in the Eastern Mediterranean
Source: Sci Rep. 2022 Mar 7;12:3662. doi: 10.1038/s41598-022-07350-w (PMC8901743; doi:10.1038/s41598-022-07350-w)
Supplement: Supplementary file 1 — Supplementary Figure S1. [file 41598_2022_7350_MOESM1_ESM.docx]

**Manuscript title:**

Structured pathways in the turbulence organizing recent oil spill events in the Eastern Mediterranean

**Author details:**

Guillermo Garcia-Sanchez. Instituto de Ciencias Matemáticas, CSIC, Madrid & Escuela Técnica Superior de Ingenieros de Telecomunicación, Universidad Politécnica de Madrid, 28040 Madrid, Spain.

Ana Maria Mancho. Instituto de Ciencias Matemáticas, CSIC, Madrid.

Antonio G. Ramos. Universidad de las Palmas de Gran Canaria, Gran Canaria.

Josep Coca. Universidad de las Palmas de Gran Canaria, Gran Canaria

Stephen Wiggins. Bristol University, Bristol

a)
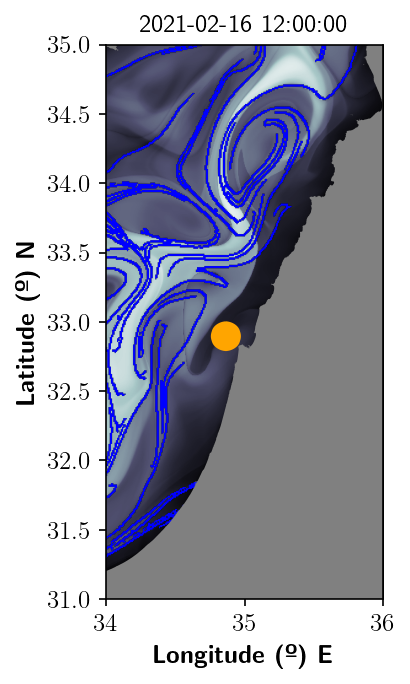
 b)
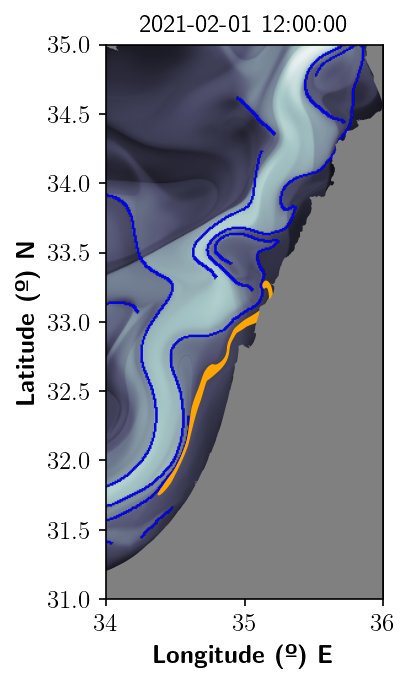


**Figure S1 legend:**

Simulations that reproduce the backward time spreading of the oil pollution on the sea. a) Representation of the pollution on the 16^th^ February 2021; b) backward time evolution on the 31st January 2021. The function M at the background is represented at times equal to the corresponding dates and the blue lines highlight the repelling material curves along which the spill aligns backwards in time. Masks indicate regions that correspond to the continental shelf. These figures have been created with Python 3.9.2 (https://www.python.org/downloads/release/python-392/). The maps shown have been generated with a mask provided by OpenStreetMaps (https://osmdata.openstreetmap.de/data/land-polygons.html).
