# Supplementary material for: Structured pathways in the turbulence organizing recent oil spill events in the Eastern Mediterranean
Source: Sci Rep. 2022 Mar 7;12:3662. doi: 10.1038/s41598-022-07350-w (PMC8901743; doi:10.1038/s41598-022-07350-w)
Supplement: Supplementary file 3 — Supplementary Legends. [file 41598_2022_7350_MOESM3_ESM.docx]

**Manuscript title:**

Structured pathways in the turbulence organizing recent oil spill events in the Eastern Mediterranean

**Author details:**

Guillermo Garcia-Sanchez. Instituto de Ciencias Matemáticas, CSIC, Madrid & Escuela Técnica Superior de Ingenieros de Telecomunicación, Universidad Politécnica de Madrid, 28040 Madrid, Spain.

Ana Maria Mancho. Instituto de Ciencias Matemáticas, CSIC, Madrid.

Antonio G. Ramos. Universidad de las Palmas de Gran Canaria, Gran Canaria.

Josep Coca. Universidad de las Palmas de Gran Canaria, Gran Canaria

Stephen Wiggins. Bristol University, Bristol

**Video legend:**

Evolution of Lagrangian Coherent Structures and of identified spills in the Eastern Mediterranean between the 25th of January 2021 and the 25th of February 2021.
